# Ability of Innate Defence Regulator Peptides IDR-1002, IDR-HH2 and IDR-1018 to Protect against *Mycobacterium tuberculosis* Infections in Animal Models

**DOI:** 10.1371/journal.pone.0059119

**Published:** 2013-03-21

**Authors:** Bruno Rivas-Santiago, Julio E. Castañeda-Delgado, Cesar E. Rivas Santiago, Matt Waldbrook, Irma González-Curiel, Juan C. León–Contreras, Jose Antonio Enciso-Moreno, Victor del Villar, Jazmin Mendez-Ramos, Robert E. W. Hancock, Rogelio Hernandez-Pando

**Affiliations:** 1 Medical Research Unit Zacatecas, Mexican Institute of Social Security-IMSS, Zacatecas, Mexico; 2 Section of Experimental Pathology, National Institute of Medical Sciences and Nutrition “Salvador Zubirán”, Mexico City, Mexico; 3 UMDNJ-School of Public Health, Department of Environmental and Occupational Health, Center for Global Public Health, Piscataway, New Jersey, United States of America; 4 Department of Immunology, School of Medicine, Autonomous University of San Luis Potosi, San Luis Potosi, Mexico; 5 Centre for Microbial Diseases and Immunity Research, University of British Columbia, Vancouver, British Columbia, Canada; Institut de Pharmacologie et de Biologie Structurale, France

## Abstract

Tuberculosis is an ongoing threat to global health, especially with the emergence of multi drug-resistant (MDR) and extremely drug-resistant strains that are motivating the search for new treatment strategies. One potential strategy is immunotherapy using Innate Defence Regulator (IDR) peptides that selectively modulate innate immunity, enhancing chemokine induction and cell recruitment while suppressing potentially harmful inflammatory responses. IDR peptides possess only modest antimicrobial activity but have profound immunomodulatory functions that appear to be influential in resolving animal model infections. The IDR peptides HH2, 1018 and 1002 were tested for their activity against two *M. tuberculosis* strains, one drug-sensitive and the other MDR in both *in vitro* and *in vivo* models. All peptides showed no cytotoxic activity and only modest direct antimicrobial activity *versus M. tuberculosis* (MIC of 15–30 µg/ml). Nevertheless peptides HH2 and 1018 reduced bacillary loads in animal models with both the virulent drug susceptible H37Rv strain and an MDR isolate and, especially 1018 led to a considerable reduction in lung inflammation as revealed by decreased pneumonia. These results indicate that IDR peptides have potential as a novel immunotherapy against TB.

## Introduction

*Mycobacterium tuberculosis* (Mtb), the cause of human tuberculosis (TB) is one of the major killers among the infectious organisms causing around 1.5 to 3 million deaths per year [1]. It has been estimated that one third of the human population carries Mtb and 10% of these people will develop active disease at some time in their lives, creating an enormous reservoir. Although the incidence of TB [1] has decreased during the past two decades, the rise of multi drug-resistant (MDR) and extensively drug-resistant (XDR) and, in the Middle East, completely resistant strains, is creating concerns regarding how to effectively treat TB infections by these recalcitrant strains [1]. In the past 40 years there has no broadly successful new Mtb drug developed. Therefore, there is a strong incentive to develop new treatments for TB and/or improve the ones currently in use to enable significant reductions in the duration of therapy and enhance patient survival. In addition to the development of new anti-tubercular drugs, immunotherapy has strong potential in treatment of this significant disease [2].

Endogenous host defence peptides are well recognized components of the innate immunity and they have been suggested to have an important role in TB infections. Such peptides can inhibit microbial growth directly through a variety of membrane and non-membrane targets [3]. However we and others have argued that their major activity involves the favourable modulation of innate immunity [2]–[6], upregulating protective immunity by mechanisms such as increasing the production of chemokines to enable the recruitment of immune cells including phagocytes, while dampening potentially harmful inflammation [3], [4]. The major groups of host defence peptides in humans are the defensins and a single cathelicidin, LL-37. It has been reported that alterations in the production of these molecules increases susceptibility to infectious diseases, including TB [7]. There are numerous reports of the immunomodulatory effects of these peptides in TB and other models [8]–[12]. Conversely, it was reported that in a murine TB model, BALB/c mice produced only low quantities of mBD-3 and mBD-4 during late progressive disease, but when these defensins were induced by the intratracheal administration of isoleucine (a defensin inducer), these animals efficiently controlled infection by both drug sensitive and drug resistant bacilli [13]. Although it seems that the use of host defence peptides would be possible for the treatment of TB, their substantial size and, for defensins, the possession disulfide bonds make their use expensive, in addition to which these peptides also have certain deleterious effects, including induction of mast cell degranulation and induction of apoptosis.

To examine the potential of immunomodulatory peptides, synthetic IDR-1 (innate defense regulator) peptide was designed to have absolutely no antimicrobial activity, but nevertheless protected against many types of bacterial infections in animal models through favorable modulation of innate immunity [14] Substitution and scrambling, and screening for enhanced ability to induce chemokines such as macrophage chemotactic protein-1 (MCP-1), led to an enhanced immunomodulatory peptide IDR-HH2 (VQLRIRVAVIRA-NH_2_) [15]. Further design based on this peptide, screening for high potency in inducing chemokines in vitro led to IDR-1002 (VQRWLIVWRIRK-NH_2_) and IDR-1018 (VRLIVAVRIWRR-NH_2_) [16], [17]. The latter two have been characterized as demonstrating an ability to protect in vivo against bacterial infections [16], [17], while IDR-1018 also significantly protected as an anti-inflammatory in a mouse model of cerebral malaria [17] and encouraged accelerated wound healing [18].

Here we evaluated the anti-infective activity of these three synthetic IDR peptides against Mtb *in vitro* and in a murine model of progressive pulmonary TB using both drug sensitive and MDR strains, demonstrating promising therapeutic efficacy.

## Results

### Characterization of in vitro Activities vs. Other Organisms

**Figure 1:**
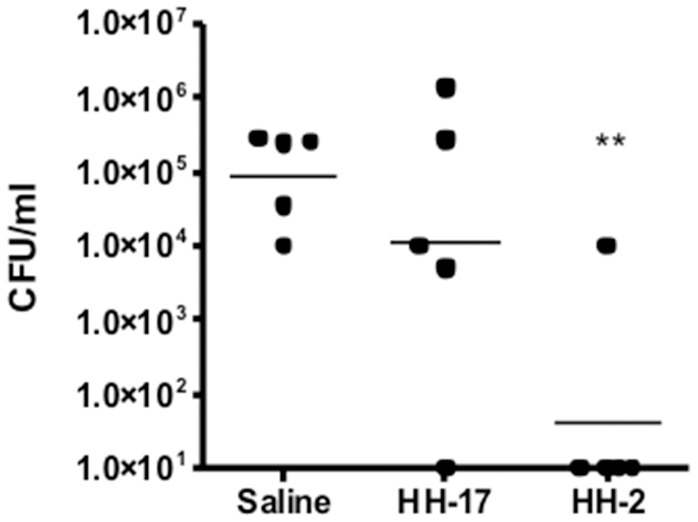
Protection by peptide HH2 against an invasive *Staphylococcus aureus* infection in mice, compared to a negative control peptide HH17. Mice were pretreated with saline (control) or 4 mg/kg peptide (IP) in saline, and infected 4 hours later with approximately 1.0×10^9^ CFU of *S. aureus*. Twenty-four hours post infection, mice were euthanized and the peritoneal lavage taken, and plated on Mueller Hinton agar. The graph is representative data from a series of 2 independent experiments. *represents p<0.05.

IDR peptides 1002 and 1018 have been previously demonstrated to have potent in vitro immunomodulatory activity [19], and good anti-infective activity in animal models (e.g. the invasive *S. aureus* model [16], [17], while IDR 1018 was shown to have modest direct antibiotic activity [19]. In contrast, HH2 has only been shown to date to have activity as a vaccine adjuvant in combination with CpG, promoting superior adaptive responses to pertussis toxoid [15]. Here we were able to demonstrate that HH2, like 1002 and 1018, induced large amounts of the macrophage/monocyte chemokines MCP-1 and Gro-α in human peripheral blood mononuclear cells (Table 1) and was protective when applied prophylactically in an invasive *Staphylococcus aureus* model in mice (Fig. 1; cf. the control peptide HH-17 - KIWVRWK-NH_2_ - that showed minimal immunomodulatory activity, Table 1). In contrast, peptides 1018, 1002 and HH2 had rather modest antimicrobial activities in vitro with MICs ranging from 5–75 µg/ml against *Pseudomonas aeruginosa* and *Staphylococcus aureus* (Table 2).

**Table 1:** Immunomodulatory activities of peptides IDR-HH2, IDR-1002 and IDR 1018 in human peripheral blood mononuclear cells (PBMC).

Cytokine	Cytokine production (pg/ml)^1^				
	No peptide	HH2	IDR-1002	IDR-1018	Control peptide HH17
MCP-1	204	5086	2676	8978	198
Gro-α	196	963	1228	1022	244
TNFα (in cells stimulated with 2 ng/ml LPS)	467	121	28	60	472

Cells were stimulated with 20 µg/ml of peptide.

1 Experiments were performed 2–4 times and means are presented. All values for peptide treated cells represent significant induction relative to the untreated control (p<0.05) for the two chemokines MCP-1 and Gro-α, and significant reduction in LPS stimulated TNF-α relative to the LPS-treated but peptide-untreated control. There was no significant induction of TNF-α by the peptides themselves. The data for IDR-1018 are consistent with those presented in Wieczorek et al [19].

**Table 2:** Direct antimicrobial activity of the IDR peptides vs. Gram negative pathogen *Pseudomonas aeruginosa* and Gram positive pathogen.

Peptide	MIC (µg/ml)	
	*P. aeruginosa*	*S. aureus*
IDR-HH2	75	38
IDR-1002	19^1^	5
IDR-1018	19	5
**HH-17**	**>50**	**>50**

*Staphylococcus aureus* - Mean of three independent experiment.

1 Taken from Wieczorek et al [19].

### Antimicrobial and Cytotoxic Activity of Synthetic Peptides

**Figure 2:**
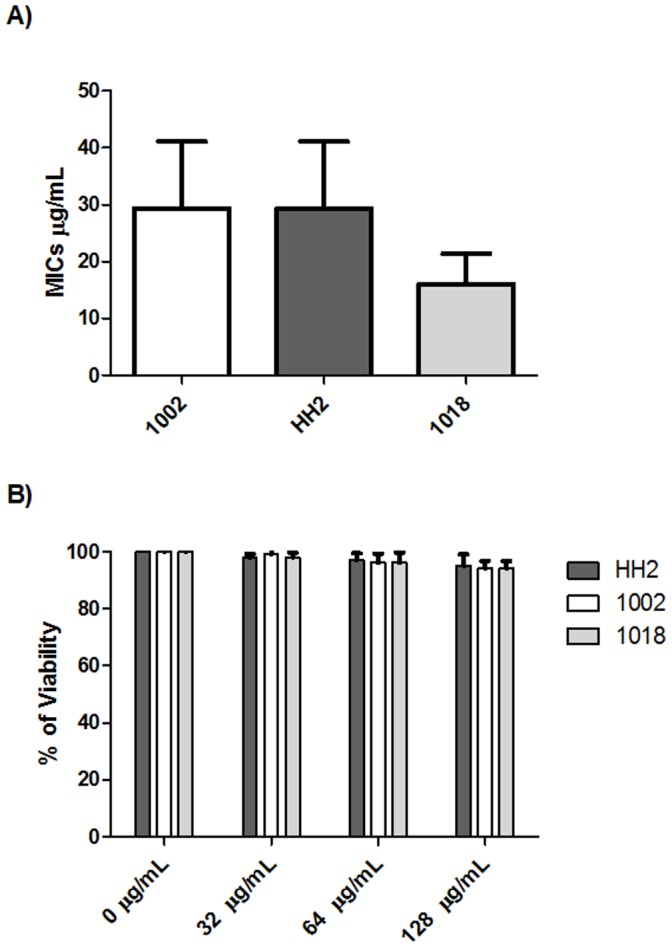
Effect of IDR peptides on the growth of *M. tuberculosis* and cytotoxicity in monocytic cells. (A) *Mycobacterium tuberculosis* strain H37Rv was incubated with increasing concentrations of the indicated peptides in doubling dilutions ranging from 128 to 8 µg/mL to determine the minimal inhibitory concentration (MIC). (B) The effect on monocyte viability was assessed by incubating increasing concentrations of these peptides with cells and assessing the integrity of the cytoplasmic membrane. The data is expressed as means ± standard deviation of 6 independent experiments with each one performed in duplicate.

The most versatile and efficient technique for preclinical testing of anti-mycobacterial drugs with direct antimicrobial activity utilizes resazurin for determining residual Mtb viability [20]. This assay was performed here to evaluate the capacity of selected antimicrobial peptides to inhibit the growth of *Mtb* strains. Figure 2A shows that all peptides had very modest antimicrobial activity against Mtb *in vitro*, with the most efficient being 1018 (MIC = 16±5.4 µg/mL), followed by 1002 and HH2 (MIC = 29.3±11.8 µg/mL). This activity was not merely due to a decrease the metabolic activity in Mtb since bacteria could not be regrown from the wells containing the lowest inhibitory concentrations we conclude that these peptides were bactericidal. Thus the direct MICs were quite weak and were intermediate between the MICs for the Gram positive pathogen *Staphylococcus aureus* and the Gram negative pathogen *Pseudomonas aeruginosa* (Table 1). To determine whether the peptides demonstrated cytotoxicity towards mammalian cells we used promonocytic cells and assessed the integrity of treated cells. There was no significant reduction in cell viability at any concentration used up to 128 µg/ml (Figure 2B); the same results were obtained using mononuclear cells from 5 different healthy donors (data not shown).

**Figure 3:**
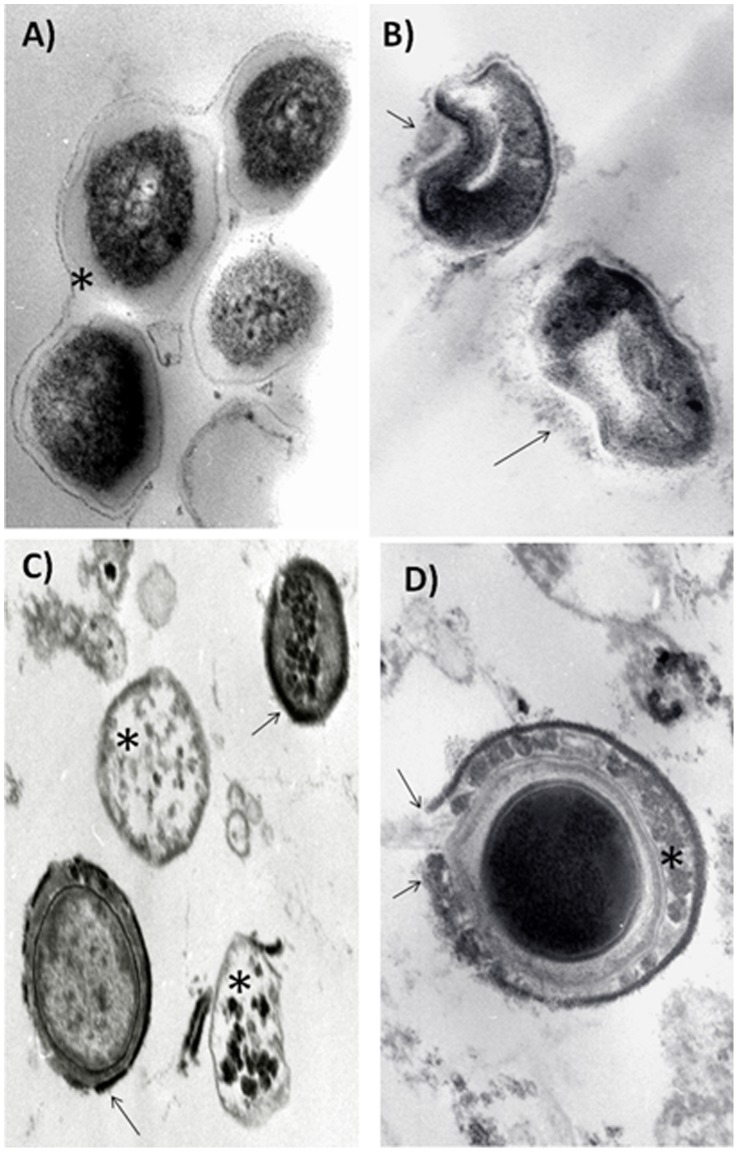
Representative electron microscopy micrographs of *Mycobacterium tuberculosis* strain H37Rv treated with high concentrations of IDR peptides. (A) Control bacteria show thick homogeneous electron lucent cell wall (asterisk) and cytoplasm with some electron dense granules (63,000x); (B) Incubation with peptide 1002 induces abnormalities in the cell wall, such as thinning, budding and partial dissolution (arrows) (100,000x). (C) Peptide HH2 induces complete disappearance of the cell wall with electron lucent cytoplasm (asterisks) or homogenous cell wall thinning and condensation (arrows) with slight electron lucent change of the cytoplasm (80,000x). (D) Incubation with peptide 1018 produced a peripheral electron dense rim with areas of rupture and dissolution (arrows) in the cell wall, which also have irregular electron dense condensations (asterisk) and an electron lucent halo around the cytoplasm (100,000x). All bars in the figures represent 5 µm. The micrographs are representative of 2 independent experiments.

Although the direct anti-bacterial activity of the IDR peptides was quite modest we examined their effects by using conventional electron microscopy. Control untreated bacilli showed a thick and well-defined homogenous and slightly electron lucent cell wall, while the cytoplasm was generally electron-dense with some medium sized granules (Fig. 3). Incubation with peptide 1002 produced substantial abnormalities in the cell wall, including thinning, budding and dissolution, while the cytoplasm showed no significant abnormalities (Fig. 3). Incubation with peptide HH2 led, in some bacilli, to an almost complete disappearance of the cell wall, with a homogenous electron-lucent cytoplasm and some electron dense granules, while other bacteria showed an electron granular appearance of the cell wall (Fig. 3). Incubation with peptide 1018 also induced significant abnormalities in the cell wall, including the formation of a peripheral electron dense rim and irregular condensations in the cytoplasmic region with mild cytoplasmic condensation which led to an electron-lucent halo between the cell wall and cytoplasm (Fig. 3). The results served to illustrate that even quite similar peptides can demonstrate significant heterogeneity in their action.

### Effect of Intratracheal Administration of IDR Peptides during Late Progressive Tuberculosis Produced by the Drug-sensitive Strain H37Rv

**Figure 4:**
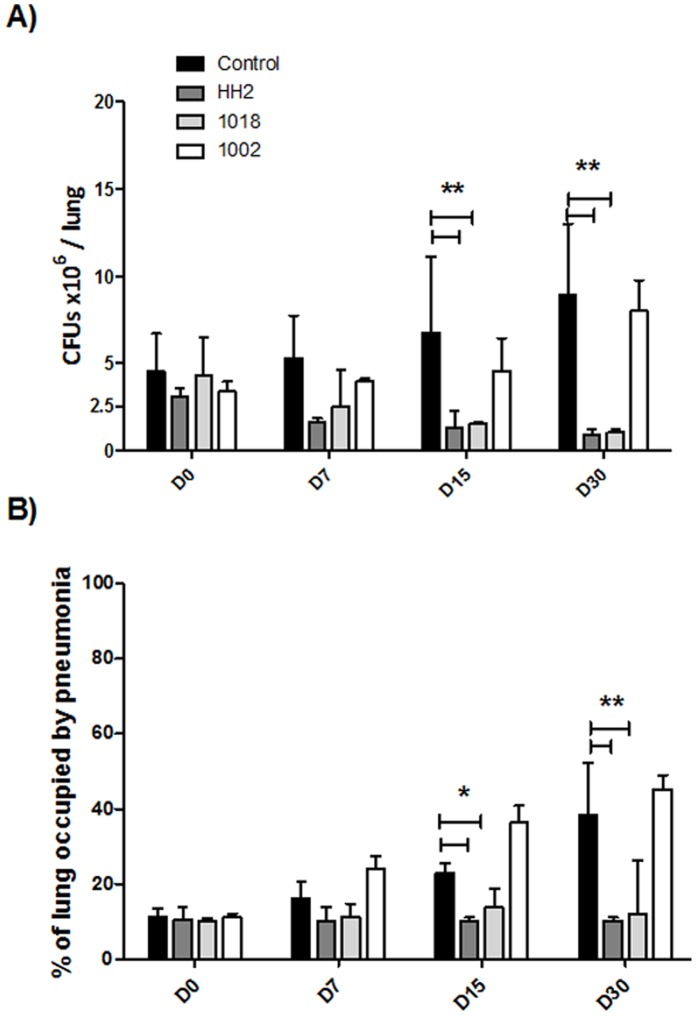
Effect of antimicrobial peptide treatment on pulmonary bacilli burdens and tissue damage (pneumonia) during experimental tuberculosis kinetic. (A) Mice were infected with the drug-sensitive H37Rv Mtb strain, and after 60 days were treated, three times per week, with ∼1 mg/kg of the indicated peptide in 100 mL of saline solution. HH2 and 1018 decreased the lung bacillary loads in comparison with untreated mice, whereas 1002 showed no significant changes. (B) Percentage of the lung surface affected by pneumonia as determined by automated morphometry. Results are expressed as means ± standard deviations, P<0.05 * or P<0.01** were considered statistically significant. Both 1018 and HH2 peptides significantly decreased the pneumonic area when compared with the control group. The graph is representative data from a series of 3 independent experiments.

**Figure 5:**
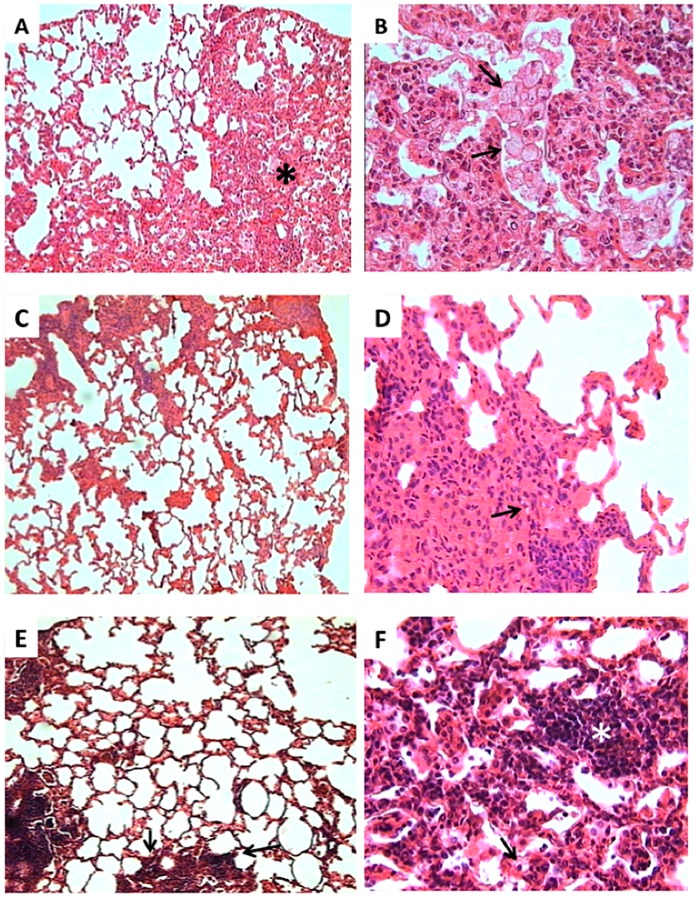
Representative lung histopathology of control non-treated and treated mice during 30 days with IDR-HH2 or IDR-1018. (A) Low power micrograph from a control mouse showing extensive pneumonic areas (asterisk) (H/E, 25x). (B) High power magnification from the same pneumonic area in control mouse showed numerous vacuolated macrophages (arrows) (H/E, 400x). (C) In contrast, the lung of mouse treated with IDR-HH2 exhibited a lesser pneumonic area (H/E, 25x). (D) High power micrograph from the pneumonic area of mouse treated with IDR-HH2 show numerous large activated macrophages with abundant and compact cytoplasm and occasional foamy macrophages (arrow) (H/E, 400x). (E) The lungs of mice treated with IDR-1018 exhibited mild pneumonia, and abundant perivascular inflammatory infiltrate, predominantly constituted by lymphocytes (arrows) (H/E, 25x). (F) High power magnification of the pneumonic area in a mouse treated with IDR-1018 showed numerous activated macrophages, occasional vacuolated macrophages (arrows) and abundant lymphocytes around blood vessels (white asterisk) (H/E, 400x). Micrographs are representative of three experiments with an n = 6.

Based on previous studies we decided to use a dose of 32 µg of each peptide in 100 µL dissolved in saline solution (∼ 1 mg/kg), which was administered intratracheally three times a week. The volume of a mouse lung is around 1 ml so, even if the entire amount of added peptide was delivered to the lung, the initial concentration of peptide in the lung would be equal to or only 2-fold higher than the MIC. Given an in vivo clearance half life (measured in the blood of mice) of 2–5 minutes (unpublished results), it seemed unlikely that any anti-infective activity would be due to direct antimicrobial activity. Treatment was started 60 days post-infection, when advanced active TB was well established [21]. During the first 15 days of 3 times-per-week treatment there was no significant reduction in bacillary loads. However at days 15 and 30 after treatment with HH2 and 1018 there was a very strong and significant reduction in bacteria, while 1002 treatment resulted in no significant reduction in bacillary loads during treatment (Fig. 4A). Consistent with these findings, after 15 and 30 days of treatment with HH2 and 1018, histological examination revealed that the lung area affected by pneumonia (assessed histologically by cellular infiltration) was significantly reduced compared to the pneumonic area in the lungs of control mice. Interestingly, not only the amount but also the cellular constitution of the inflammatory infiltrate was different (Fig. 5); the pneumonic areas in control non-treated mice showed numerous foamy vacuolated macrophages surrounded by numerous lymphocytes (Fig. 5A, 5B), while the pneumonic areas from treated mouse with HH2 showed abundant activated macrophages (large cells with abundant compact cytoplasm and big nucleus with peripheral marginated chromatin and prominent nucleolus), and lesser amount of lymphocytes (Fig. 5C, 5D); the pneumonic areas from mice treated with 1018 also showed predominant activated macrophages and thick inflammatory cuffs predominantly constituted by lymphocytes around blood vessels and airways (Fig. 5E, 5F). Mice treated with 1002 showed non-significant differences in lung area affected by pneumonia (Fig. 4B).

### Effect of Intratracheal Administration of IDR Peptides during Late Progressive Tuberculosis Produced by a Multidrug-resistant Strain

**Figure 6:**
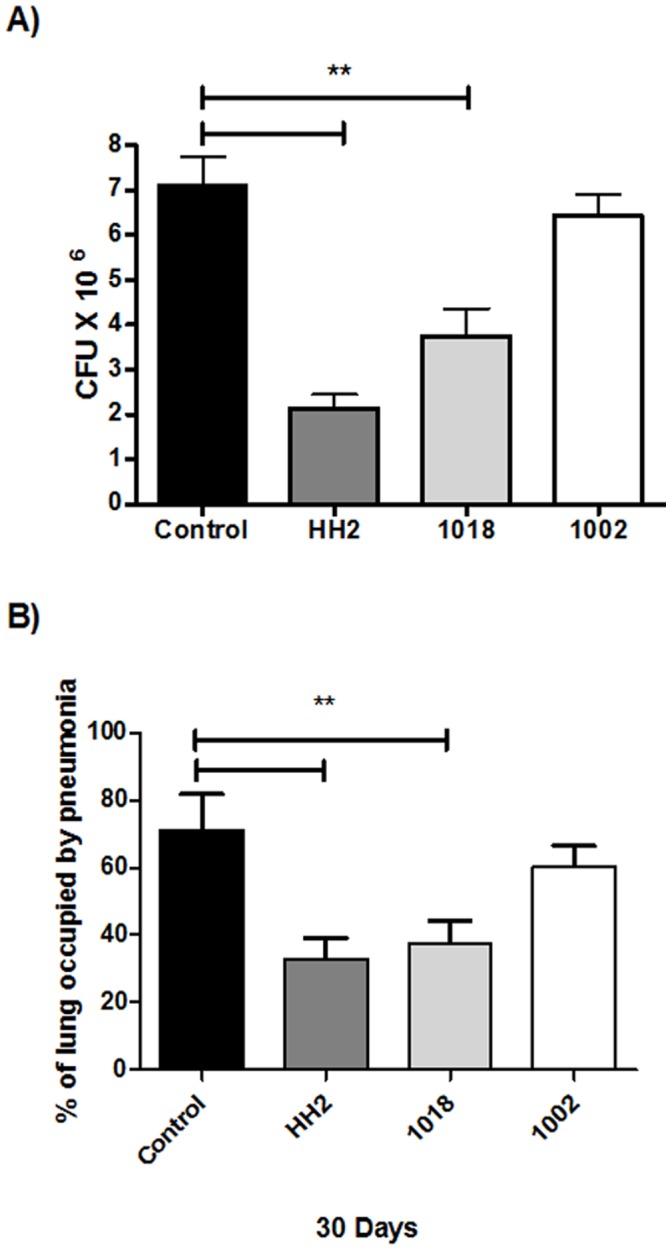
Effect of antimicrobial peptides on the treatment of mice infected with a drug-resistant strain. (A) Animals were infected with the MDR strain and after 60 days treated three times per week with 32 µg in 100 µL of saline solution of the indicated peptide. Compared to the control untreated animals, IDR peptides HH2 and 1018 induced a significant decrease in lung bacillary loads. (B) Measurement of the area of consolidation of pneumonia showed that only HH2 and 1018 significantly reduced the pneumonic areas when compared with control mice. The data is expressed as means ± standard deviation of 3 independent experiments; p<0.01 indicated with a bar and **over the two bars was considered statistically significant.

MDR-infected mice treated with either HH2 or 1018 demonstrated a significant 3 to 5-fold reduction in CFU counts, whereas 1002 showed no statistically significant difference compared to the non-treated mice group (Fig. 6A). The same trend towards activity in decreasing the pneumonic area was seen, with HH2 and 1018 showing statistical significance in reducing the pneumonic area, also with numerous activated macrophages and less frequent foamy macrophages, while treatment with peptide 1002 showed a modest but non-significant reduction when compared to mice that received only saline solution (Fig. 6B).

## Discussion

The present study demonstrates that two different IDR peptides showed notable anti-infective activity in an animal model against both a drug-sensitive and MDR strain (Fig. 4,5). Since the peptides used in this study belong to the IDRs that were developed as modulators of innate immunity [14]–[17], we sought to determine whether these peptides had meaningful antimicrobial effects against *M. tuberculosis*. As shown by the results of *in vitro* experiments, all peptides had marginal direct antimicrobial activity (Fig. 2A) with MICs of 16–30 µg/ml. Indeed our electron microscopy study, which showed disruption, thinning and budding of the bacterial cell wall after the incubation of bacilli with these peptides suggested that these peptide directly damaged bacterial integrity (Fig. 3). Nevertheless, the peptides were active in vivo at intratracheally-delivered doses of only 32 µg/mouse (∼1 mg/kg), and thus would be unlikely to reach concentrations in the lung equivalent to their in vitro MICs (due to binding to tissues, and rapid clearance). Thus it seems very unlikely that these peptides were acting as direct antimicrobials in our in vivo mouse models. Similarly we can discount general induction of host cell damage since none of the peptides used in this study were cytotoxic to host cells (Fig. 2B). While we cannot discount the possibility that direct antimicrobial activity was contributing to the in vivo anti-tuberculosis effect, it seems likely that this was due to the immunomodulatory effects of the IDR peptides, that include the induction of chemokines promoting recruitment of cells that would act against the bacilli, the promotion of differentiation of these cells into more effective bacterial-killing cells, the suppression of excessive pro-inflammatory cytokine production and the enhancement of adaptive responses, all of which have been shown for IDR peptides [14,16,17,19; Table 1]. Intriguingly the two active peptides, IDR-HH2 and IDR-1018 were also considerably more potent at inducing the monocyte/macrophage chemokine MCP-1 in vitro, cf. inactive IDR-1002 (Table 1). However we cannot exclude that differential activity related to another property of these peptides such as differential pharmacokinetics in animals or differential synergy with immune changes that occurred due to the Mtb infection [22], [23].

We previously reported that BALB/c mice infected by the intratracheal route with a high dose of the drug-sensitive H37Rv strain, showed an early high production of antimicrobial peptides and Th1 cytokines, which together with high levels of TNF-α were proposed to temporarily control the infection. After 4 weeks of infection, there is a decrease in the levels of antimicrobial peptides (mainly defensins), as well as IFN-γ and TNF-α. Gradually, pneumonic area prevails over granulomas. Extensive pneumonia plus a high burden of bacteria causes death [24]. Thus, we initiated treatment with the IDR peptides after 8 weeks of infection, when active disease was occurring, mimicking a common clinical situation in developing countries. The intratracheal instillation of the IDR peptides HH2 and 1018 led to decreased lung bacillary loads after 15 and 30 days of treatment, although the peptide 1002 did not show significant decrease in bacillary loads (Fig. 4), even though all 3 peptides showed similar in vitro immunomodulatory (and antimicrobial) effects (Tables 1,2). We have observed substantial heterogeneity in the in vivo properties of the different peptides. For example IDR-1 protects in the invasive *Staphylococcus aureus* model but not in a cerebral malaria model [14], while IDR-1018 protects in both models [17]. This indicates that there may be subtle, or even profound, differences in the immunomodulatory properties of individual peptides. Consistent with this, the two protective peptides 1018 and HH2 also considerably decreased the percentage of pneumonic area, consistent with an anti-inflammatory activity [15,17,19; Table 1]. Moreover, the cellular constitution of the inflammatory infiltrate in the pneumonic areas was also different; control mice showed predominant foamy macrophages which are characteristic of late disease in this model. Foamy macrophages are large cells with numerous cytoplasmic vacuoles, abundant phagocytosed bacilli and high expression of molecules that efficiently suppress cellular protective immunity such as transforming growth factor or prostaglandin E (24). Interestingly, the pneumonic areas from mice treated with IDR-HH2 or 1018 showed occasional foamy macrophages and abundant activated macrophages which are quite similar to the predominant phagocyte type during early infection in this model and contribute to the efficient control of bacilli growth by the expression of antibacterial factors such as TNF or iNOS [24]. With regards to the MDR strain, our results (Fig. 5) showed similar trends to those seen for the drug-sensitive Mtb strain; HH2-and 1018 were able to reduce lung bacillary loads and percentage of pneumonic area, as seen before while 1002 showed no improvement in either of these parameters.

In conclusion, our results show that repeated intrapulmonary administration of HH2 and 1018 IDR peptides leads to efficient suppression of the growth of tuberculosis bacilli and reduction of the pneumonic area in an experimental model of pulmonary TB. This occurred when mice were infected with either drug-sensitive or drug-resistant mycobacteria using a high dose model of infection (2–5×10^5^) in Balb/c mice treated with the given therapeutic regime. Further experiments are needed to determine if these peptides could be used as prophylactic strategy for household contacts who are constantly exposed to mycobacteria, however prior data has shown for other bacteria that the peptides act prophylactically when added prior to bacteria exposure [14], [16]. Nevertheless, since this study showed the efficacy of these peptides in mice, they are candidate for translation to use in humans.

## Materials and Methods

### Ethics Statement

All the animal work was done according to the guidelines and approval of the Ethical Committee for Experimentation in Animals of the National Institute of Medical Sciences and Nutrition in Mexico (permit: cinva 268), in accordance with Mexican national regulations on Animal Care and Experimentation (NOM 062-ZOO-1999).

### Peptide Synthesis and Design

Peptides IDR-1002 (VQRWLIVWRIRK-NH_2_), IDR-1018 (VRLIVAVRIWRR-NH_2_) and IDR-HH2 (VQLRIRVAVIRA-NH_2_) were synthesized using F-moc chemistry at the Nucleic Acid/Protein Synthesis Unit (University of British Columbia, Vancouver, British Columbia, Canada) or at GenScript (Piscataway, NJ). Peptides were HPLC purified to >95% purity and confirmed by mass spectrometry. None of the peptides had any endotoxin contamination based on Limulus amoebocyte lysate assay or inability to induce TNF-α in human peripheral blood mononclear cells. Peptides were generally resuspended in water or 0.9% saline to a stock concentration of 4 mg/ml on the day of use.

### *Mycobacterium Tuberculosis* Strain Growth

Drug-sensitive Mtb strain H37Rv (ATCC no.25618) and MDR strain (clinical isolate, resistant to first line antibiotics) were grown in Middlebrook 7H9 broth (Difco Laboratories, Detroit, MI, USA) supplemented with 0·2% (v/v) glycerol, 10% oleic acid, albumin, dextrose and catalase (OADC enrichment media, BBL, Becton Dickinson, Franklin Lakes, New Jersey ) and 0·02% (v/v) Tween-80 at 37°C. Mid log-phase cultures were used for all experiments. For *in vivo* studies, mycobacteria were counted and stored at –80°C until use. Before use, mycobacteria aliquots were thawed and pulse-sonicated to remove clumps.

### Microdilution Colorimetric Reduction Assay

Susceptibility testing utilizing resazurin as an indicator of residual bacterial viability (St. Louis, MO, USA) was performed in 96-well flat-bottom plates (Corning., NY, USA) as described previously [20]. Briefly, all test wells contained 100 µL of 7H9-OADC supplemented growth media. One hundred µL of the diluted peptide at the highest concentration starting at 128 µg/mL were added to one well, the contents of these wells were mixed thoroughly and 100 µL transferred into the next well; the process was then repeated, thus creating serial two fold-dilutions. In addition to the tested peptides, rifampin (8 µg/mL) was used as positive control, and medium without any compound was used as negative control in each plate. One hundred µL of a 1∶25 dilution of the Mcfarland standard (OD_600 nm_ = 0.769) for each Mtb strain was added into each well. Peptides were tested in the concentration range of 8 to 128 µg/mL.

Plates were placed in a plastic bag and incubated at 37°C for 5 days. On day 5, 20 µL of 0.01% resazurin solution (Trek Diagnostic, Westlake, OH, USA) and 12 µL of sterile 10% Tween 80 solution were added to several control wells containing Mtb but no antibacterial agent and incubated again for 24 hours under the same conditions. If the Mtb viability controls tested positive for resazurin reduction (as indicated by a pink colour), resazurin was added to all wells. The minimal inhibitory concentration (MIC) was defined as the lowest peptide concentration that prevented the reduction of resazurin and therefore a color change from blue to pink. Previous studies by our group suggest that some host defence peptides may induce dormancy or a bacteriostatic state in *M. tuberculosis* [22]. To examine this, 10 µl from the lowest concentration that did not reduce resazurin were serially diluted and seeded onto 7H10 agar plates supplemented with Middlebrook OADC enrichment media and incubated for at least 21 days at 37°C, to observe if Mtb regrowth occurred.

MICs for *Pseudomonas aeruginosa* strain H103 and *Staphylococcus aureus* ATCC#25923 were determined by the broth microdilution assay as described previously [20].

### Cell Culture, Cytotoxicity Assay and in vitro Immunomodulatory Activity

The human *U937* promonocytic *cell* line was obtained from American Type Culture Collection (ATCC), and maintained in RMPI, supplemented with 10% FBS (Sigma), 1% 5,000 units/mL penicillin, 5,000 µg/mL streptomycin (Sigma). The cells were incubated in an atmosphere of 5% CO_2_ at 37°C with different concentrations of the different peptides with variable concentrations from 128 to 8 µg/mL for 18 h. All cells used in this study were between passages 5 and 15. Subsequently to incubation, cells were mixed with Guava Viacount reagent and allowed to stain for 10 minutes (Guava Technologies, Hayward, CA). The Guava System differentiates viable from non-viable cells by detecting fluorescence signals from two fluorescent DNA-binding dyes: one membrane-permeable dye stains all nucleated cells while the second dye enters cells when membrane integrity has been compromised, ie, non-viable cells. Viable cells were quantified using a Guava Personal Analyzer (PCA) flow cytometer (Guava Technologies), according to the manufacturer’s specifications.

Peripheral blood mononuclear cells were prepared from fresh human blood collected from health volunteers under human ethics approval from the University of British Columbia, as described previously [16]. Cytokine induction assays and assessments of the reduction of LPS-induced TNFα were performed exactly as described previously [16].

### Protection by HH2 in an Animal Model of Invasive S. aureus Disease

Studies were performed exactly as described previously [16], [17] in accordance with UBC animal ethics approval. Mice were injected IV with 8 mg/kg of peptide in saline or an equivalent volume of saline solution 4 hours prior to initiation of an invasive Staphylococcus aureus infection administered intraperitoneally.

### Experimental Model of Progressive Pulmonary TB in BALB/c Mice

Animal work was performed in accordance with Mexican national regulations on Animal Care and Experimentation (NOM 062-ZOO-1999). The experimental model of progressive pulmonary TB was described in detail elsewhere [23]. Briefly, male BALB/c mice, 6–8 weeks of age, were anaesthetized in a gas chamber using 0·1 mL per mouse of sevofluorane, and each mouse infected by endotracheal instillation with 2·5×10^5^ live bacilli. Mice were maintained in the vertical position until they spontaneously recovered. Infected mice were maintained in groups of five in cages fitted with micro-isolators.

### Treatment of the Infected Mice with IDR Peptides

After 60 days of infection, animals were allocated arbitrarily into four groups. Peptide treatment started 60 days after infection, when advanced progressive disease was well established. We used a dose of 32 µg in 100 µL of saline solution (∼1 mg/kg) for the therapeutic experiments, performing three independent experiments. All groups of animals received the corresponding dose three times a week for up to 4 weeks by intratracheal instillation, since preliminary studies indicated no efficacy via the intraperitoneal delivery route. Six animals in each group were sacrificed at 7, 15 and 30 days after starting treatment. The efficiency of the each peptide treatment was determined by quantifying the lung bacillary loads by assessing colony-forming units (CFU) as described below, and assessing the extent of tissue damage by histopathology.

### Determination of CFU in Infected Lungs

One lung from each of three mice at each time-point was used to determine CFU while the other lung was used for histology analysis as described below. Lungs were homogenized with a polytron device (Kinematica, Lucerne, Switzerland) in sterile tubes containing 1 mL 0·05% Tween-80 in PBS. Five dilutions of each homogenate were spread onto duplicate plates containing Bacto Middlebrook 7H10 agar (Difco) enriched with oleic acid, albumin, catalase and dextrose-enriched medium (Becton Dickinson, Sparks, MD, USA). The number of colonies was counted after 21 days of incubation at 37°C with 5% CO_2_.

### Preparation of Lung Tissue for Histology

The lungs from each of three different animals per time point and group were perfused intratracheally with ethyl alcohol (J:T: Baker, Mexico City, Mexico). Lungs were then dehydrated and embedded in paraffin (Oxford Labware, St Louis, MO, USA), sectioned and stained with haematoxylin and eosin. The percentages of the lung surfaces affected by pneumonia were determined using an automated image analyzer (Axiovert m200, Carl Zeiss, Germany).

### Ultrastructural Analysis of Treated *Mycobacterium tuberculosis*

The determination of the ultrastructural damage to *Mycobacterium tuberculosis* caused by the treatment with the different IDR peptides was evaluated using transmission electron microscopy. Briefly, bacilli were cultured in Middlebrook 7H9 broth (Difco, Detroit, MI) supplemented with Middlebrook OADC enrichment media (BBL, BD, Franklin Lakes, New Jersey) until logarithmic phase was achieved. Viable bacilli (1×10^7^) were placed in the wells of 96 well plates and exposed to the corresponding IDR peptides (128 µg/mL, each) for 18 hours at the MICs determined previously using the resazurin assay. Subsequently, bacteria were fixed for two hours with 4% glutaraldehyde dissolved in cacodilate buffer. A second fixation was performed with osmium tetraoxide fumes. Then, bacteria were dehydrated and embedded in epon resin. Thin sections were mounted in cooper grids, stained with uranyl acetate and lead citrate and observed with a Zeiss M-10 electron microscope (Carl Zeiss, Overkochen, Germany).

### Statistical Analysis

The data was analyzed by parametric two way ANOVA with Tukeýs post-test or a non-parametric Kruskal Wallis multiple comparisons test with Dunn’s post-test. The Graphpad 5.02 software (Graph Pad Inc, La Jolla, California) was used to perform the analysis. For all analysis a P value <0.05 was considered statistically significant.
